# Chondrogenic and BMP-4 primings confer osteogenesis potential to human cord blood mesenchymal stromal cells delivered with biphasic calcium phosphate ceramics

**DOI:** 10.1038/s41598-021-86147-9

**Published:** 2021-03-24

**Authors:** Meadhbh Á. Brennan, Mario Barilani, Francesco Rusconi, Julien de Lima, Luciano Vidal, Cristiana Lavazza, Lorenza Lazzari, Rosaria Giordano, Pierre Layrolle

**Affiliations:** 1 Inserm, UMR 1238, PHY-OS Laboratory, Bone Sarcomas and Remodelling of Calcified Tissues, Faculty of Medicine, University of Nantes, Nantes, France; 2 Laboratory of Regenerative Medicine-Cell Factory, Department of Transfusion Medicine and Hematology, Fondazione IRCCS Ca’ Granda Ospedale Maggiore Policlinico, Milan, Italy; 3 Present Address: National University of Ireland (NUIG), Galway, Ireland; 4 Present Address: Rapid Manufacturing Platform, GEM Laboratory, Centrale Nantes, Nantes, France

**Keywords:** Biomedical engineering, Stem cells, Mesenchymal stem cells, Multipotent stem cells, Stem-cell differentiation, Stem-cell research

## Abstract

Bone marrow mesenchymal stem/stromal cells (BMSCs) show great promise for bone repair, however they are isolated by an invasive bone marrow harvest and their regenerative potential decreases with age. Conversely, cord blood can be collected non-invasively after birth and contains MSCs (CBMSCs) that can be stored for future use. However, whether CBMSCs can replace BMSCs targeting bone repair is unknown. This study evaluates the in vitro osteogenic potential of unprimed, osteogenically primed, or chondrogenically primed CBMSCs and BMSCs and their in vivo bone forming capacity following ectopic implantation on biphasic calcium phosphate ceramics in nude mice. In vitro, alkaline phosphatase (intracellular, extracellular, and gene expression), and secretion of osteogenic cytokines (osteoprotegerin and osteocalcin) was significantly higher in BMSCs compared with CBMSCs, while CBMSCs demonstrated superior chondrogenic differentiation and secretion of interleukins IL-6 and IL-8. BMSCs yielded significantly more cell engraftment and ectopic bone formation compared to CBMSCs. However, priming of CBMSCs with either chondrogenic or BMP-4 supplements led to bone formation by CBMSCs. This study is the first direct quantification of the bone forming abilities of BMSCs and CBMSCs in vivo and, while revealing the innate superiority of BMSCs for bone repair, it provides avenues to induce osteogenesis by CBMSCs.

## Introduction

Autologous bone grafting remains the gold standard method employed clinically to heal large bone defects, with bone being the second most transplanted tissue, after blood. However, repair of bone defects continues to be a significant clinical challenge since autologous bone grafting is compromised by the limited quantity and quality of donor bone tissue and the associated pain and morbidity. The development of mesenchymal stromal cell (MSC) therapy offers an alternative to autologous bone grafting, whereby MSCs are delivered to the defect site on a biomaterial scaffold. MSCs were first isolated from the bone marrow (BMSC)^1^, and as such are the most studied and characterized, however MSCs have also since been extracted from other sources including adipose tissue^2^ and the umbilical cord^3^. Presently, BMSCs are the primary cell source in bone tissue engineering strategies since they have shown to effectively orchestrate bone formation and healing in pre-clinical animal studies^4–6^ and in clinical trials^7–9^. However, the isolation of BMSCs from the posterior iliac crest is an invasive procedure, the concentration of MSCs in the bone marrow is extremely low, thus requiring several weeks of expansion in Good Manufacturing Practices (GMP) facilities prior to cell therapy applications, and the capacities of BMSCs have been shown to decline with age and disease, thereby limiting their use in several circumstances.

Human cord blood mesenchymal stromal cells (CBMSCs) can been isolated from the blood of umbilical cords after birth^3^, which is completely safe to both mother and baby. Extensive characterization of CBMSCs reveals that they display the typical phenotypic and tri-lineage potential of stromal cells^10–12^. In particular, CBMSCs present osteogenic differentiation capacity in vitro^10,13–15^. The gold standard method to investigate the inherent osteogenic properties of cells in vivo is the ectopic bone formation test, because the lack of naturally residing osteogenic cells allows the assessment of the exclusive ability of the transplanted cells to induce de novo bone by either directly differentiating into bone forming cells or by the recruitment of host progenitor cells to the site which ultimately form the new bone tissue. CBMSCs that have undergone osteogenic priming prior to transplantation have demonstrated enhanced mineralization and bone regeneration in ectopic sites^16^ and critical-sized bone defects^17^ respectively. Taken together, these evidences suggest that CBMSCs may be a possible alternative to BMSCs for bone regeneration since they exhibit osteogenic properties, avoid the invasive BM harvesting procedure, and can be banked at birth for later use. However, while unprimed BMSCs consistently form bone after transplantation^4–6^, whether CBMSCs have this capacity remains largely unexplored. Furthermore, while chondrogenic priming of BMSCs and adipose tissue MSCs has been shown to be an effective strategy to modulate their bone forming potential via endochondral ossification^18–22^, whether this might also be effective for CBMSCs has not yet been investigated.

The aim of this study was to investigate the bone formation potential of human CBMSCs in comparison to BMSCs. First, the in vitro osteogenic differentiation was evaluated. Bone formation and neovascularization by transplanted human BMSCs and CBMSCs and their engraftment in vivo were then evaluated in an ectopic mouse model. Moreover, to establish whether priming of CBMSCs is a prerequisite prior to implantation, BMSCs and CBMSCs were transplanted after priming with standard osteogenic supplements, bone morphogenetic protein-4 (BMP-4), or chondrogenic differentiation.

## Results

### Morphological, phenotypic and tri-lineage differentiation characterization of MSCs

**Figure 1:**
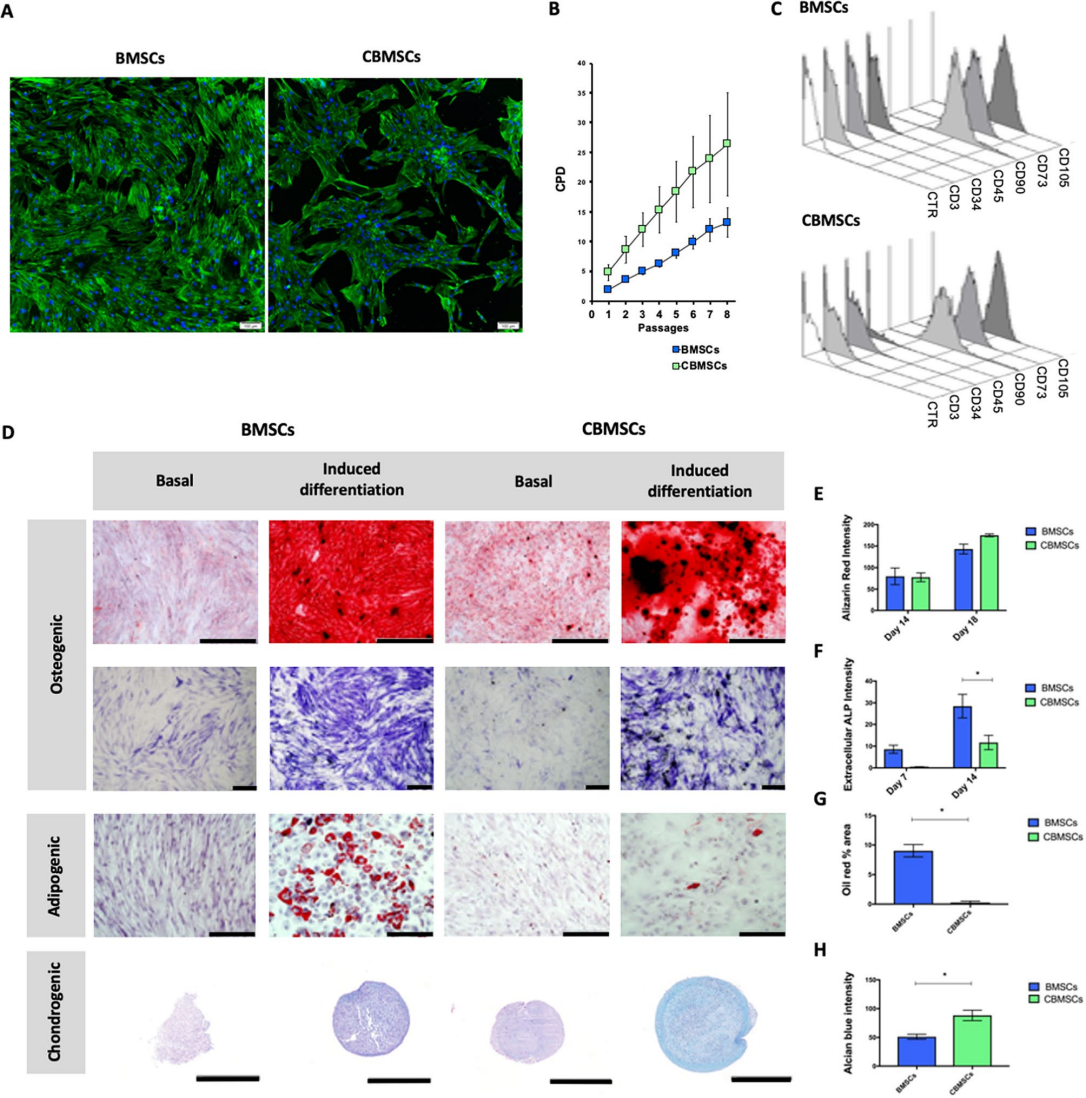
Characterization of BMSCs and CBMSCs. (**A**) Cell morphology as illustrated by DAPI nuclear, and phalloidin actin cytoskeleton fluorescent staining. Scale bars represent 200 μm. (**B**) Growth curve is represented as Mean ± standard deviation (SD) of cumulative population doublings (CPD) for passage 1–8. (**C**) Phenotypic characterization by flow cytometry of BMSCs and CBMSCs shows positive expression of stem cell surface markers CD105, CD90, CD73, and negative expression for CD45, CD34, and CD3. (**D**) Representative images of in vitro tri-lineage differentiation potential of BMSCs and CBMSCs towards osteogenic, adipogenic, and chondrogenic lineages confirmed by histochemical staining with Alizarin red and Alkaline phosphatase, Oil red O, and Alcian blue, with scale bars representing 500 μm, 200 μm, 200 μm, and 500 μm, respectively. (**E**) Quantitative measurement of solubilized bound Alizarin red stain of both BMSCs and CBMSCs induced with osteogenic supplements after 7 and 14 days. (**F**) Extracellular ALP staining quantification after 7 and 14 days of osteogenic priming. (**G**) Quantification of lipid droplet area in images of oil red staining following adipogenic induction of BMSCs and CBMSCs. (**H**) Quantification of Alcian blue colour intensity of sections through pellets of BMSCs and CBMSCs that underwent chondrogenic priming. Data is presented as Mean ± standard error of the mean (SEM). Statistical analysis was by unpaired Student’s *t*-test for single experimental condition (**B**, **G**, **H**) and by two-way ANOVA followed by Holm–Sidak’s multiple comparison test for multiple experimental conditions (**E**, **F**). *Significant difference between MSCs of different tissue origin.

MSCs from both bone marrow and cord blood show a long spindle-like morphology, as depicted in Fig. 1A. While BMSCs grew homogeneously distributed in a monolayer, CBMSCs consistently formed clusters or colonies of cells. Intra-passage population doubling time during exponential growth phase was 17.3 ± 1.8 h for BMSCs and 10.5 ± 2.6 h for CBMSCs (p = 0.08976, Student’s *t*-test). CBMSCs also showed fast inter-passages growth kinetics compared with BMSCs, as showed by lower cumulative population doublings over passages, although statistical significance was not reached (p > 0.05 for each passage, Student’s *t*-test) (Fig. 1B).

Phenotypic profiles, as assessed by flow cytometry, were similar for both BMSCs and CBMSCs as presented in Fig. 1C. Both BMSCs and CBMSCs showed positive expression of typical stem cell surface markers CD105, CD90, CD73, while they were negative for CD45, CD34, and CD3. Both BMSCs and CBMSCs displayed tri-linage differentiation potential towards the osteogenic, adipogenic, and chondrogenic lineages, as observed in Fig. 1D, albeit to sharply varying degrees. In terms of extracellular matrix mineralization, as assessed by Alizarin red staining, there was no difference between BMSCs and CBMSCs at either time point, as depicted in Fig. 1D,E. Extracellular ALP expression was significantly higher for BMSCs compared with CBMSCs at both days 7 and 14 (Fig. 1D,F). BMSCs exhibited large lipid droplets after 21 days of adipogenic priming, whereas CBMSCs adipogenic potential was extremely limited and significantly lower (Fig. 1D,G). CBMSCs exhibited significantly superior tendency for chondrogenic differentiation when induced with chondrogenic supplements in pellet culture for 21 days compared with BMSCs as shown in Fig. 1D,H, indeed even in the absence of chondrogenic priming, CBMSCs pellets cultured in basal media spontaneously produced glycosaminoglycans.

### Cell growth, intracellular ALP activity and cytokine secretion of primed MSCs

**Figure 2:**
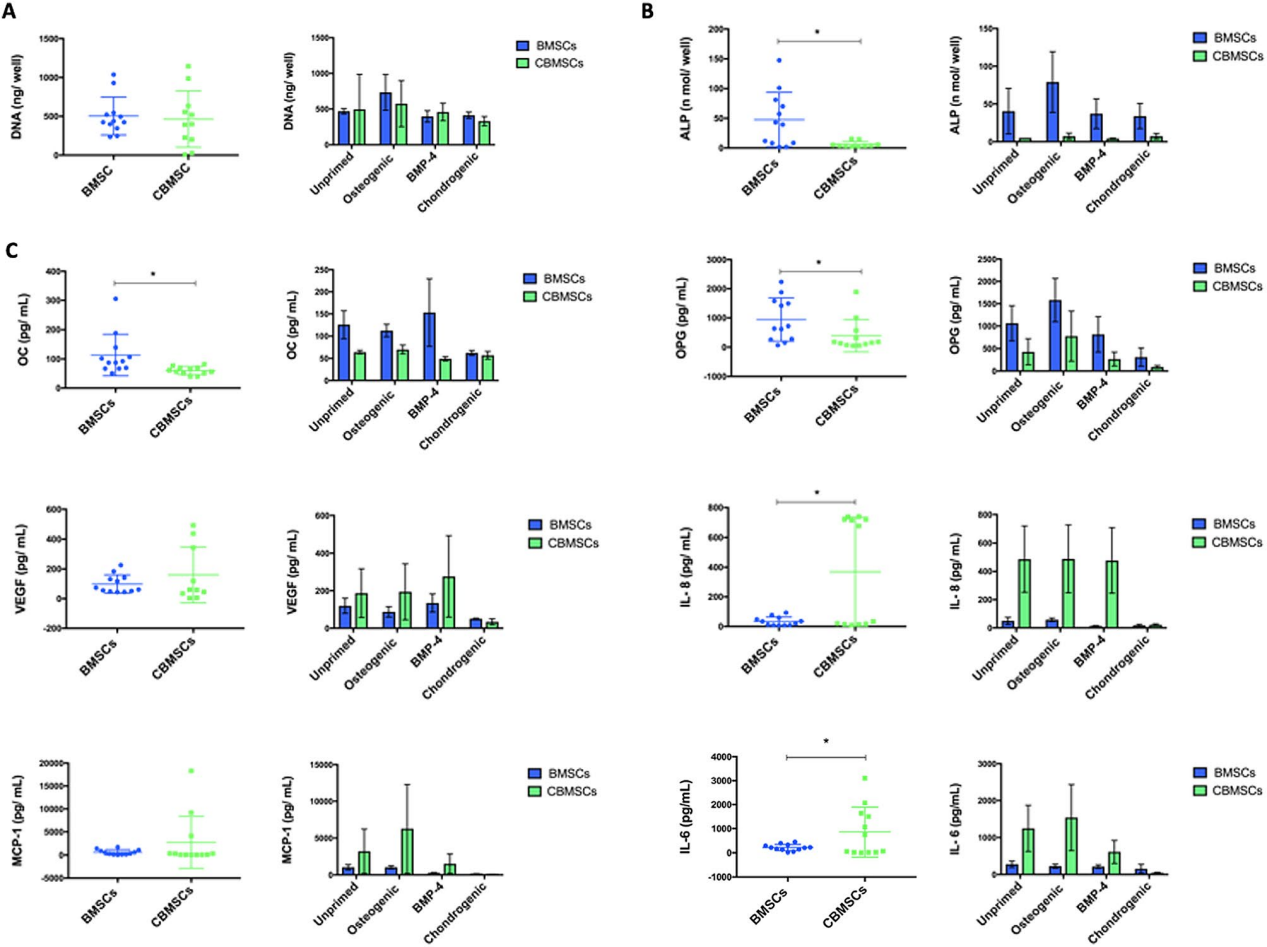
Growth, differentiation and secretion profiles of BMSCs and CBMSCs after priming with either standard osteogenic differentiation supplements, BMP-4, or chondrogenic supplements. Scatter plots represent all MSC donor samples pooled regardless of priming, while bar charts present Mean ± SEM of each priming condition. *Significant difference between MSC origin. (**A**) Cell proliferation after 6 days of priming as assessed by Pico Green Assay for DNA content. (**B**) Intracellular ALP expression after 6 days of priming. (**C**) Multiplex array analysis of soluble secreted osteogenic cytokines; Osteoprotegerin (OPG), Osteocalcin (OC), and Osteopontin (OPN), immunogenic cytokines; Interleukin 6 and 8 (IL-6,8), Monocyte chemoattractant protein 1 (MCP-1), as well as angiogenic factors; Vascular Endothelial Growth Factor (VEGF)*.* Statistical analysis was by unpaired Student’s *t*-test for single experimental condition (dot plots) and by two-way ANOVA followed by Holm–Sidak’s multiple comparison test for multiple experimental conditions (histograms). *Significant differences between MSCs of different tissue origin.

Cell proliferation of both BMSCs and CBMSCs during the 6 days of either osteogenic or chondrogenic priming prior to implantation was measured by the pico-green assay which quantifies DNA content. As shown in Fig. 2A, there was no difference in the growth rate between MSCs of different origins, or as a consequence of priming in this time period (6 days). Intracellular alkaline phosphatase was significantly enhanced in BMSCs compared to CBMSCs as shown in Fig. 2B. As depicted in Fig. 2C, osteoprotegerin (OPG) and osteocalcin (OC) secretion were significantly higher in BMSCs compared to CBMSCs, Interleukin (IL)-6 and IL-8 were significantly higher in CBMSCs compared with BMSCs, while VEGF and MCP-1 were secreted at similar levels, as revealed by using Bio-Plex multiplex arrays. Chondrogenic priming of both BMSCs and CBMSCs reduced secretion levels of OPG, IL-6, IL-8, MCP-1, and VEGF, but no significant differences were observed.

### Gene expression of primed MSCs

**Figure 3:**
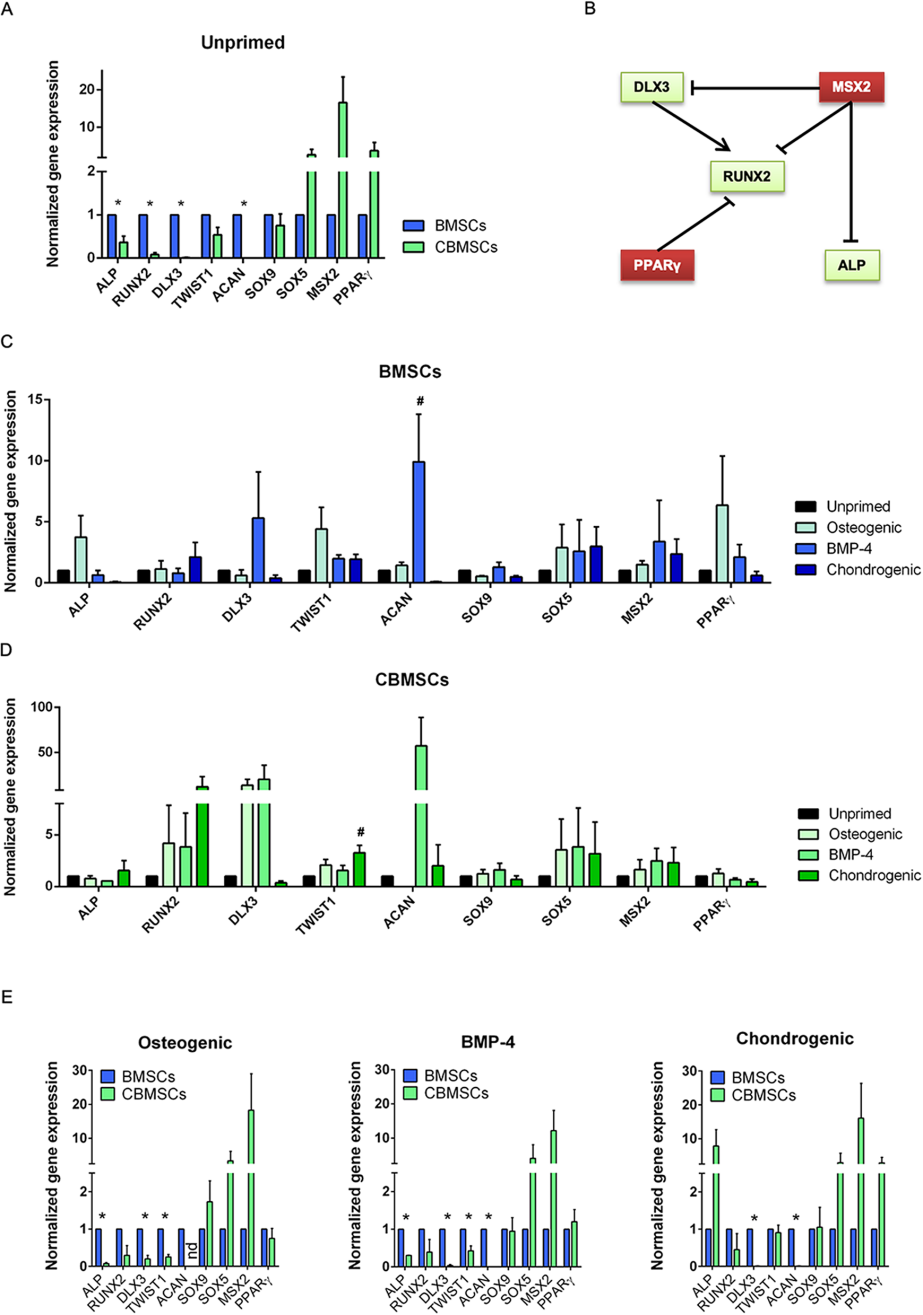
Gene expression profiles of unprimed and primed BMSCs and CBMSCs. (**A**) Histogram showing gene expression of BMSCs and CBMSCs at the steady state; values are represented as Mean ± SEM and normalized to BMSCs. (**B**) Schematic summarizing positive and negative regulations of the gene expression of crucial osteogenic and chondrogenic players. (**C**) Histogram representing transcriptional profile of BMSCs upon different differentiation primings protocols; values are represented as Mean ± SEM and normalized to unprimed BMSCs. (**D**) Histogram representing transcriptional profile of CBMSCs upon different differentiation primings protocols; values are represented as Mean ± SEM and normalized to unprimed CBMSCs. Statistical analysis was by unpaired Student’s *t*-test for single experimental condition (**A**, **E**) and by one-way ANOVA followed by Holm–Sidak’s multiple comparison test for multiple experimental conditions (**C**, **D**). *Significant difference between MSCs of different tissue origin (**A**, **E**); ^#^significant difference between primed and unprimed MSCs (**C**, **D**).

Gene expression of key regulators of osteogenic and chondrogenic differentiation pathways was addressed by qRT-PCR. Unprimed BMSCs showed significantly higher expression of genes associated with osteoblast differentiation (Student’s *t*-test: p < 0.05 for ALP, RUNX2, DLX3; p = 0.0536 for TWIST1) than unprimed CBMSCs (Fig. 3A). SOX9 was expressed at similar levels, whereas SOX5, MSX2 and PPARγ were more expressed in unprimed CBMSCs than BMSCs, although statistical significance was not reached. This could be indicative of a molecular state less prone to readily undergo osteogenic differentiation for CBMSCs, because MSX2 and PPARγ are negative regulators of ALP, RUNX2 and DLX3 transcription (Fig. 3B).

Upon priming, BMSCs showed a trend of increased expression for ALP, TWIST1 and SOX5 (osteogenic priming), DLX3, ACAN, SOX5 (BMP-4 priming), RUNX2 and SOX5 (chondrogenic priming) (Fig. 3C), although only BMP-4 priming-induced upregulation of ACAN gene expression reached statistical significance. Most notably, PPARγ and MSX2 showed a trend of increased expression upon priming (Student’s *t*-test, p > 0.05 for both genes), which could hamper osteogenic differentiation of primed BMSCs, as described above for unprimed CBMSCs.

On the other hand, priming of CBMSCs induced a trend of increased expression for RUNX2, TWIST1, SOX5 (all primings), DLX3 (osteogenic and BMP-4 priming) and ACAN (BMP-4 priming) (Fig. 3D). Intriguingly, chondrogenic stimuli showed higher RUNX2 increase, concomitant to higher induction of ALP and TWIST1, and marked reduction of PPARγ. Statistical significance was reached only by chondrogenic priming-induced upregulation of TWIST1 (p < 0.05, Student’s *t*-test). This change in transcript levels could lead to increased osteogenic differentiation, following again the schematic described above (Fig. 3B).

Direct comparison between BMSCs and CBMSCs revealed that osteogenic and BMP-4 primings levelled the gene expression trends previously observed between unprimed BMSCs and CBMSCs for what concerns PPARγ, but not ALP (Fig. 3E). Conversely, chondrogenic priming rescued the significant differences detected in unprimed condition for ALP, RUNX2 and TWIST1 gene expression (Fig. 3E). In particular, the latter priming induced a tenfold upregulation of ALP transcript level in CBMSCs compared with BMSCs. Altogether, these data point at a possible advantageous effect of the applied primings on CBMSC osteogenic potential.

### Gene expression and secretion properties of MSCs upon attachment to biomaterial

A standard operating procedure was previously developed to mix BMSCs with the BCP biomaterial, which allowed BMSCs to attach to the scaffold over the course of 1 h, prior to implantation^4,23^. While cell attachment is enhanced with further incubation time (with maximal attachment after 4 h), a 1 h attachment duration was previously chosen^7,23^, since this time frame is appropriate in the surgical clinical setting. As observed by both scanning electron microscopy and methylene blue staining (Supplementary Fig. 1), this procedure also achieved attachment and even distribution of both BMSCs and CBMSCs onto the surface of the BCP granules prior to implantation.

**Figure 4:**
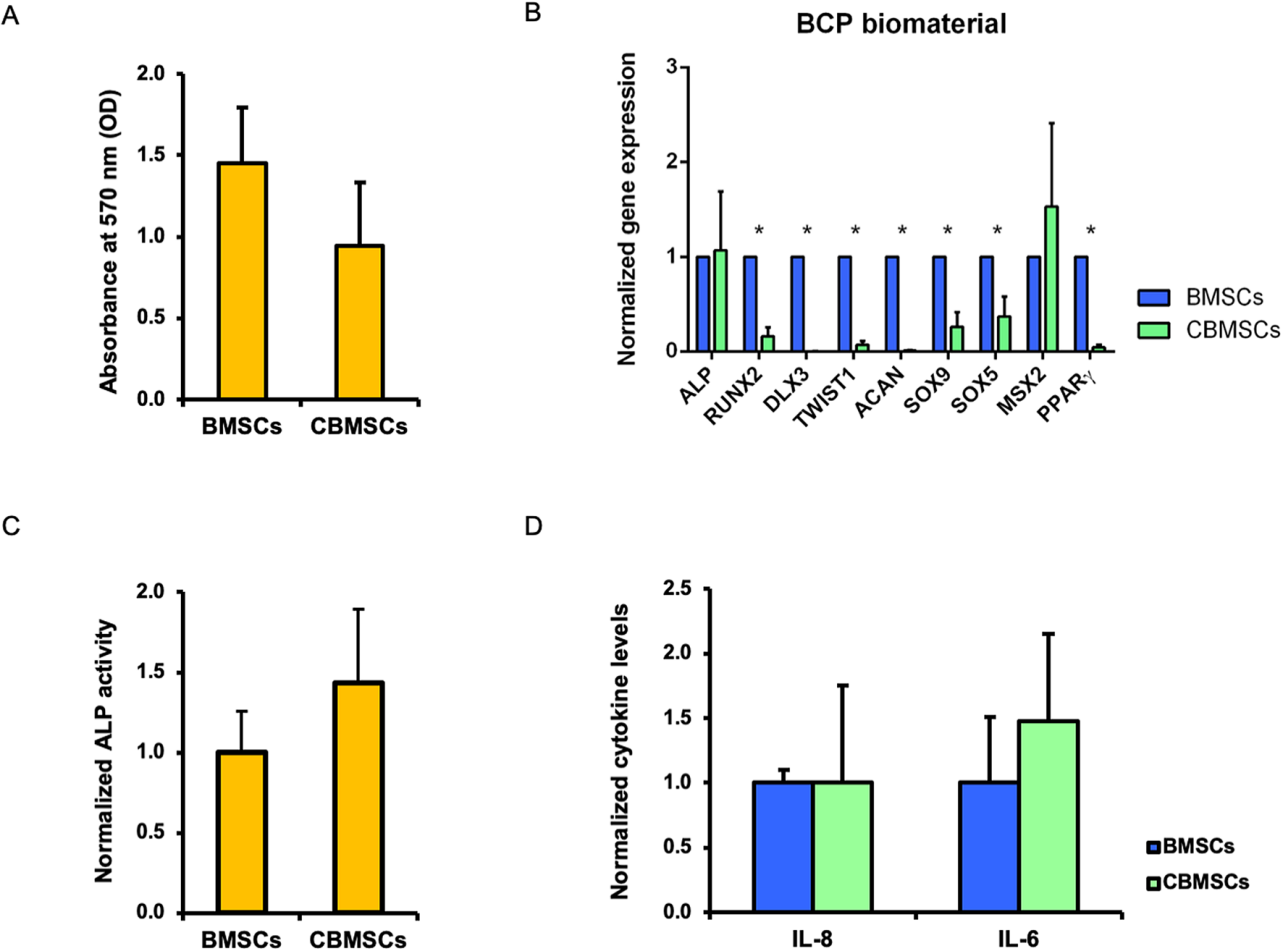
Gene expression profiles and secretion properties of BMSCs and CBMSCs cultured on BCP biomaterial. (**A**) Histogram showing optical density (OD) at 570 nm as Mean ± standard deviation (SD). (**B**) Histogram showing gene expression of BMSCs and CBMSCs as Mean ± standard error of the mean (SEM) and normalized to BMSCs. (**C**) Histogram showing ALP activity as Mean ± SD and normalized to BMSCs. (**D**) Histogram showing levels of secreted interleukin (IL)-8 and IL-6 as Mean ± SD and normalized to BMSCs. Statistical analysis was by unpaired Student’s *t*-test. *Significant difference between MSCs of different tissue origin.

To investigate any influence of BCP biomaterial on the osteogenic induction of unprimed BMSCs and CBMSCs before in vivo testing, longer culture conditions (6 days) were applied to BCP-MSC 3D constructs, after which gene expression by qRT-PCR and secretion properties by enzymatic assay or ELISA were analysed. First, cell proliferation was assessed by MTT assay. All cells proliferated onto the BCP biomaterial, showing similar (p = 0.22, Student’s *t*-test) growth properties (Fig. 4A). Next, the same panel of genes previously analysed for the differentiation priming 2D experiments was implemented to evaluate the transcriptional profile of BCP-MSC 3D constructs. Intriguingly, osteogenic ALP gene expression was rescued in CBMSCs, matching BMSC level (p = 0.9125, Student’s *t*-test), whereas negative regulator of osteogenesis PPARγ was significantly downregulated (Fig. 4B). Significant differences were also detected for osteogenic RUNX2, DLX3 and TWIST1, and for chondrogenic ACAN, SOX9 and SOX5. A decreasing trend was observed also for MSX2, which showed similar expression levels for BMSCs and CBMSCs (Fig. 4B) compared to the tenfold upregulation observed in 2D unprimed conditions for CBMSCs (Fig. 3A).

Extracellular ALP activity was also addressed. Consistent with the gene expression data, CBMSCs and BMSCs showed similar enzymatic activity of secreted ALP (p = 0.2296, Student’s *t*-test) (Fig. 4C), completely rescuing the significant differences observed in the 2D setting for unprimed cells (Fig. 2B). Secretion properties of BCP-MSC 3D constructs were further investigated to determine cytokine extracellular levels. The statistical difference observed in the 2D setting (Fig. 2B) was not observed after culture of cells on BCP biomaterial for both IL-8 (p = 0.9996, Student’s *t*-test) and IL-6 (p = 0.3821, Student’s *t*-test) (Fig. 4D).

### Unprimed CBMSCs are not osteogenic but priming induces osteogenesis in vivo

**Figure 5:**
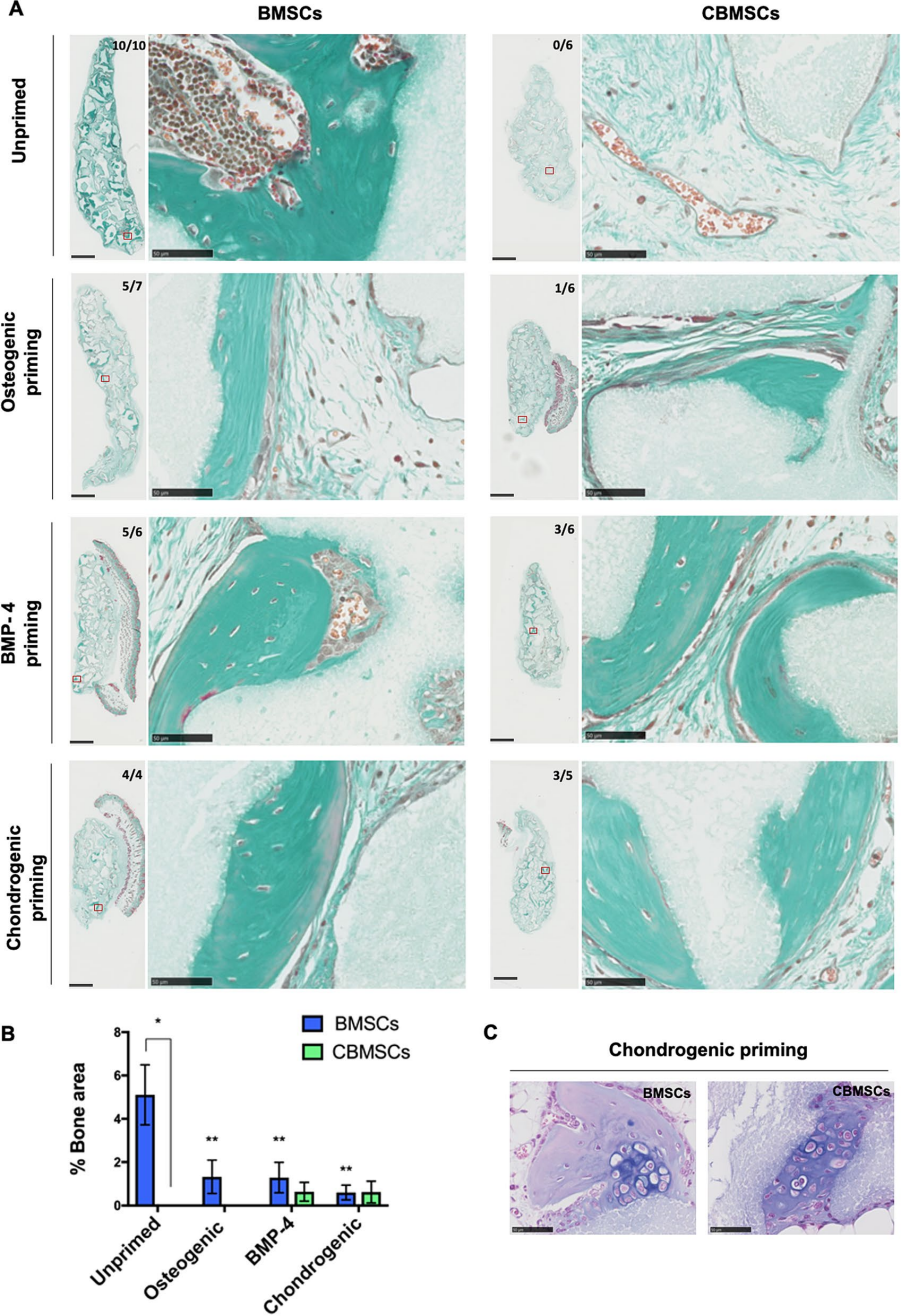
Ectopic bone formation by implantation of BCP biomaterial in combination with either BMSCs or CBMSCs, w/o priming prior to implantation in nude mice. (**A**) Masson’s trichrome staining of explanted tissue sections after 8 weeks. Newly formed bone is evidenced in green, while BCP biomaterial is shown in grey. The bone incidence score on each group represents the number of implants with newly formed ectopic bone over the total number of implants in that group. The red box on the whole implant sections represents each magnified image presented. Scale bars represent 1 mm and 50 μm for whole implants and magnified images respectively. (**B**) Histomorphometry quantification of ectopic bone formed in each group. Data are presented as mean ± SEM of the mean. (**C**) Alcian blue staining of explants after 8 weeks in subcutis sites. Cartilage was observed in both BMSC and CBMSC groups, but only when cells underwent chondrogenic priming prior to implantation. Statistical analysis was by two-way ANOVA followed by Holm–Sidak’s multiple comparison test. *Significant difference between MSCs of different tissue origin; **statistical difference between unprimed and primed conditions of BMSCs.

BMSCs or CBMSCs w/o prior osteogenic or chondrogenic priming were implanted with BCP biomaterials into subcutaneous sites in nude mice. These were the same bone marrow and cord blood donor MSCs that were used for the in vitro studies. Figure 5A shows cross sections through samples eight weeks after implantation. Masson’s trichrome staining shows bone in green and BCP biomaterial in grey, with quantification of bone area presented in Fig. 5B. While abundant bone tissue and mature bone marrow territories were observed with unprimed BMSCs (bone induction: 10/10 implants), unprimed CBMSCs did not induce any bone formation (0/6 implants). Interestingly, priming with both BMP-4 (3/6 implants) or chondrogenic factors (3/5 implants) prior to implantation imparted bone formation potential to CBMSCs, and priming with standard osteogenic supplements only yielded bone formation in 1/6 implants in minute quantities. Furthermore, unprimed BMSCs achieved significantly more (p < 0.05) bone formation compared to any of the priming conditions. Strikingly, cartilage tissue, identified by Alcian blue staining, was observed exclusively in samples with MSCs that underwent chondrogenic priming prior to implantation, for both BMSCs and CBMSCs, and this was often surrounded by newly formed bone tissue (Fig. 5C).

### Neovascularization by transplanted MSCs

**Figure 6:**
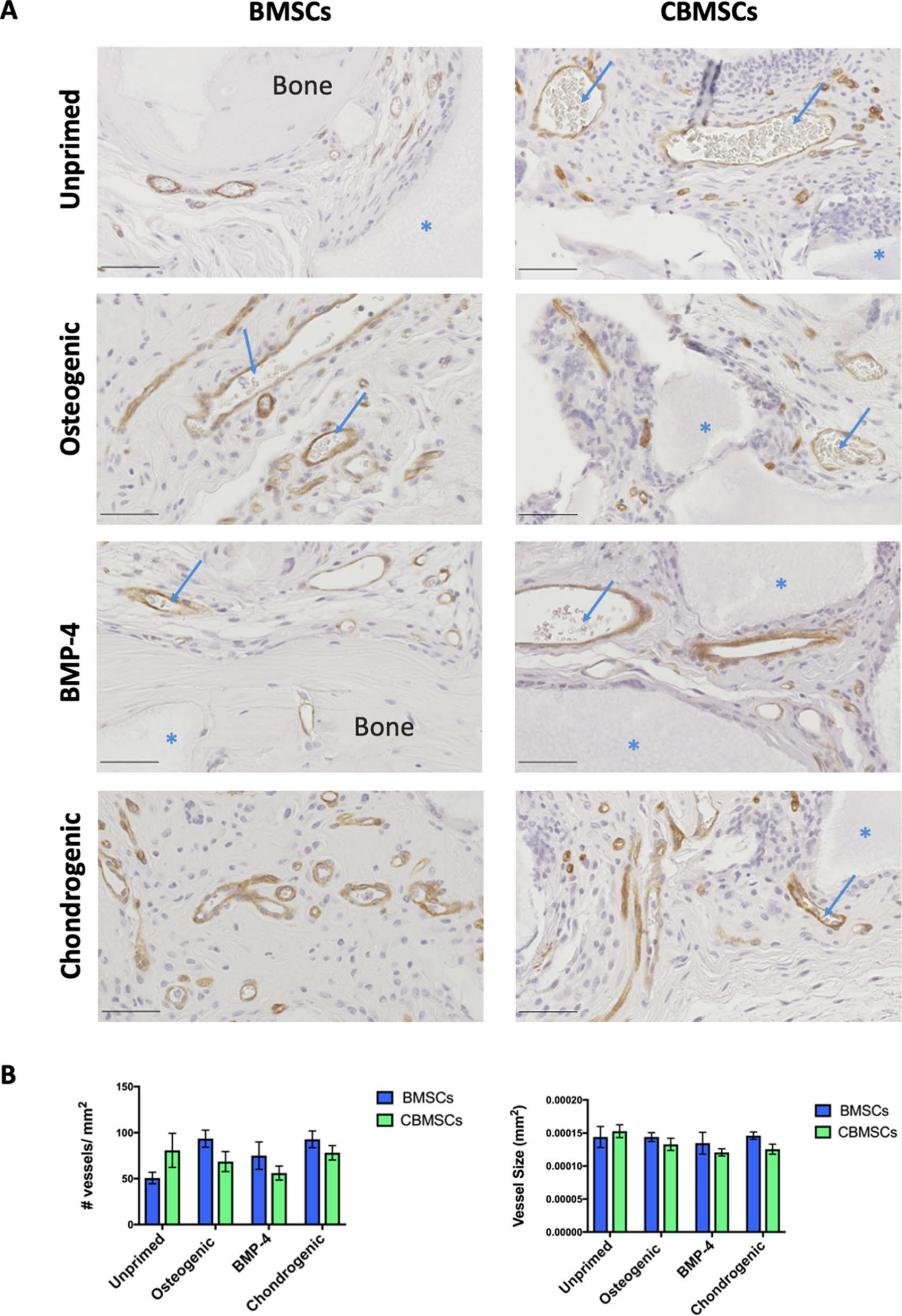
CD146 immunostaining for neovascularization. (**A**) Blood vessels are shown in brown with erythrocytes inside the blood vessels highlighted by the blue arrows, newly formed bone tissue is indicated and BCP biomaterials are shown in grey as indicated by the blue asterisks. Scale bars represent 50 μm. (**B**) Quantification of the number of blood vessels per mm^2^, and the size of newly formed blood vessels of BMSC and CBMSC groups ± priming. Data are presented as mean ± SEM. Statistical analysis was by two-way ANOVA followed by Holm–Sidak’s multiple comparison test.

After 8 weeks of implantation in vivo, both BMSCs and CBMSCs samples appeared well vascularized as illustrated in Fig. 6A, showing CD146 immunostaining with blood vessels in brown. While there was a trend towards an increased number of blood vessels in sections of unprimed CBMSC samples compared to their BMSC counterparts, this did not reach statistical significance. Furthermore, there was no difference in the number of blood vessels between different priming groups. No difference in the size of blood vessels was observed either according to MSC origin, or between different priming groups (Fig. 6B).

### Engraftment of human MSCs in host mice

**Figure 7:**
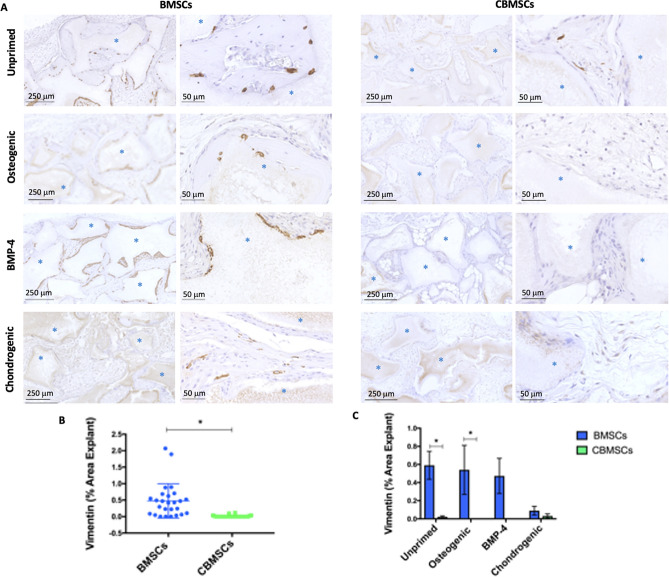
Engraftment of human MSCs in vivo by immunohistochemistry for Vimentin. Human MSCs are observed in brown along the periphery of BCP biomaterials or embedded in osteocyte lacunae dispersed throughout the newly formed bone matrix. The majority of cells present are of mouse host origin shown by the blue/purple nuclei. (**A**) Engraftment is shown in BMSC and CBMSC samples 8 weeks after transplantation of unprimed cells, cells primed with standard osteogenic supplements, cells primed with BMP-4, and cells primed with chondrogenic supplements (blue asterisks indicate the BCP granules). (**B**) The quantification of Vimentin identification of human cells for all implants, (**C**) together with consideration for different priming regimes prior to implantation. Data are presented as mean ± SEM. Statistical analysis was by unpaired Student’s *t*-test for single experimental condition (dot plot) and by two-way ANOVA followed by Holm–Sidak’s multiple comparison test for multiple experimental conditions (histogram). *Significant difference between MSCs of different tissue origin.

Immunohistochemistry for Vimentin was used to determine engraftment of transplanted human MSCs in mice after 8 weeks as depicted in Fig. 7. Human cells were found primarily along the periphery of the BCP biomaterials and within osteocyte lacunae in the bone matrix as observed in the unprimed BMSC group, in contrast to the unprimed CBMSC group that only had a minute quantity of engrafted cells dispersed within the fibrous tissue. Engrafted cells were also observed in the BMSC group that underwent priming with osteogenic supplements, BMP-4, or chondrogenic supplements. There were significantly more implants containing human cells 8 weeks after implantation in the BMSC group (20/25), compared with the CBMSC group (6/25). When the percentage area of Vimentin staining was quantified per implant in all groups, it was observed that it was significantly higher (p < 0.0001) in BMSC explants compared to the CBMSC group (Fig. 7B). With regard to the effect of priming conditions on eventual MSC survival after 8 weeks in vivo, there were 100%, 86%, 40%, and 75% of implants transplanted with BMSCs containing human cells in the unprimed, osteogenic, BMP-4, and chondrogenic groups respectively. For CBMSCs, these same priming conditions led to 50%, 0%, 0%, and 20% of implants containing human cells respectively. It was observed that the area of Vimentin staining was significantly higher in the unprimed (p = 0.0089) and the osteogenic primed (p = 0.0467) BMSC group compared to the CBMSC group (Fig. 7C).

## Discussion

Repair of large bone defects caused by trauma, bone tumor resection, or metabolic bone diseases continues to be a challenge in orthopedic surgery because of the various disadvantages of autologous bone grafting^24^. Advances in tissue engineering strategies including mesenchymal stromal cells (MSCs) offer promising alternatives and the identification of appropriate tissue sources for cell therapy is an important task in regenerative medicine. Owing to their in vitro growth and differentiation potentials, and imperatively their ease of accessibility and safety of collection, MSCs derived from umbilical cord blood (CB) are an interesting option for cell therapy. Furthermore, the use of CB as source of cells for therapeutic purposes offers many advantages, such as low risk of transmission of infections and environmental contaminants, low immunogenicity, and abundance of raw material at international networks of public cord blood banks^25^. The current study assessed the potential of CBMSCs for bone tissue regeneration, in particular their osteogenesis in direct comparison with the gold standard bone marrow-derived MSCs (BMSCs). There were significant differences in the in vitro soluble secretion profiles, gene expression, and osteogenic differentiation capacity, while in vivo, significant differences in the capacity of MSCs to generate ectopic bone according to their tissue of origin were observed*.* Priming regimens of various differentiation induction factors revealed that both BMP-4 and chondrogenic priming imparted in vivo bone forming capacity to CBMSCs. The contribution of MSC culture on BCP biomaterial was also investigated in vitro and showed a similar modulation of the same molecular pathways elicited by priming conditions.

While both BMSCs and CBMSCs displayed similar spindle-like morphologies, it was consistently observed that when cells began to reach confluency, CBMSCs grew in clusters, unlike the homogenously dispersed BMSCs. The growth rates of the MSCs from both origins were comparable, as were the typical phenotypic profiles of ‘stromal cell’ surface markers. Both BMSCs and CBMSCs possessed tri-lineage capacities in vitro, albeit to varying degrees. Osteogenic differentiation as measured by ALP staining was significantly higher in BMSCs. There was a striking lack of adipogenic differentiation of CBMSCs, unlike BMSCs, consistent with previous observations^10,11,26–28^. A stark difference in the chondrogenic potential of BMSCs and CBMSCs was observed in vitro, whereby significantly higher Alcian blue staining was observed in CBMSC compared to BMSC pellets. This has not been reported previously in the typical chondrogenic pellet tri-lineage protocols, however it is in line with recent observations that CBMSCs form cartilage in vitro that is more histologically and mechanically equivalent to native cartilage compared to that formed by BMSCs^29^ and that unprimed CBMSCs formed significantly higher quantities of cartilage in vivo compared with BMSCs in a ceramic-based assay similar to the current study^30^. Together, these suggest that CBMSCs may be superior for cartilage regeneration applications compared with BMSCs, which warrants further investigation.

ALP, both at the gene expression level, as well as intracellular and extracellular protein level, was found to be significantly elevated in BMSCs compared with CBMSCs in the current study, in agreement with a recent in vitro study^31^. Interestingly, it was observed that MSX2, which has been shown to suppress ALP transcription at the promoter level and to antagonize osteoblast differentiation^32,33^, was up-regulated in CBMSCs compared to BMSCs. Since ALP has been shown to be a marker of bone healing in patients^7^, this may represent an important difference between the two MSC sources in terms of their osteogenesis. Intriguingly, CBMSC ALP gene expression was rescued by chondrogenic priming and in vitro culture on BCP biomaterial. In addition to ALP, expression of other osteogenic-related genes such as RUNX2 and DLX3 and the secretion of cytokines which induce osteogenesis, such as OPG and OC, were higher in BMSCs compared with CBMSCs and together these may contribute to the observed significantly elevated bone formation capacity of BMSCs compared to CBMSCs in vivo. Interestingly, all primings effectively levelled significant differences observed in unprimed cells for RUNX2. To note, negative regulator of osteogenesis PPARγ^34,35^ was more expressed in CBMSCs than BMSCs and dramatically reduced by priming regimens and, more consistently, by culture of MSCs on BCP biomaterial. The opposite modulation of crucial positive (RUNX2, ALP) and negative (MSX2, PPARγ) regulators of osteogenesis could explain the beneficial effect exerted on CBMSCs and the detrimental effect on BMSCs, even though this hypothesis would need tailored mechanistic validation.

A direct quantitative comparison of the bone forming potential of BMSCs and CBMSCs has not been reported previously. However, the bone formation capacity of CBMSCs in vivo after osteogenic priming with standard supplements and lack of osteogenicity without priming observed here is consistent with previous reports in critical sized bone defects in nude mice^17^.

The current study is the first to show the osteoinductive potential of CBMSCs as a consequence of chondrogenic priming, or indeed the administration of BMP-4. Interestingly, both the aforementioned priming conditions were superior compared to priming with standard osteogenic supplements (dexamethasone, ascorbic acid, beta-glycerolphosphate), that previously showed mineralization^16^ and bone formation by CBMSCs^17^. While the current study does not decipher the underlying mechanisms that impart bone forming potential to CBMSCs as a consequence of BMP-4 or chondrogenic priming, several potential factors should be addressed. First, the immunomodulatory secretions, in particular IL-6, IL-8 and MCP-1, of CBMSCs, which were significantly higher compared to BMSCs in unprimed conditions, were vastly reduced by chondrogenic priming. BMP-4 reduced the secretion of IL-6 and MCP-1, however a clear demonstration of a causative relationship of these phenomena has not been given. Nevertheless, an important interplay between transplanted BCP biomaterials and MSCs and their immunomodulatory influence on bone regeneration has been established in previous studies^36^. Increased production of inflammatory cytokines by CBMSCs was addressed in the literature and linked to a possible physiological role in the establishment and modulation of an immunologically dynamic environment at the fetal–maternal interface for proper implantation of the blastocyst, development of the embryo and delivery of the fetus^37^. Furthermore, the mechanism of bone formation by BMP-4 and chondrogenically primed MSCs were distinct. As observed by Alcian blue staining, cartilage tissue was identified in tissue sections, but only in the groups where the MSCs had undergone chondrogenic priming. This suggests that bone tissue was formed by endochondral ossification in this group, while no cartilage was found in any of the other groups, or at any earlier time points in our previous studies, thus indicating that bone tissue was formed by an intramembraneous ossification in the other groups. This shows that although disparate routes were taken by chondrogenic and BMP-4 primed MSCs, both were capable of initiating tissue regeneration in vivo. MSCs of passages 4–5 were utilized in the current study and this might affect their bone forming abilities in vivo^6^. Significantly more human BMSCs engrafted in host mice compared with CBMSCs. Interestingly, more BMSCs were observed in the unprimed group compared with the primed groups, which paralleled the degree of bone formation. In the CBMSC group, no increase in cell engraftment paralleled increased bone formation. These data seem to hint at two different mechanisms occurring in our in vivo ectopic bone formation model, that are (1) direct formation of bone tissue by transplanted BMSCs and (2) recruitment and stimulation of endogenous osteoprogenitors^38–40^. Both direct^6^ and indirect paracrine participation^41,42^ of transplanted MSCs in bone regeneration have been suggested and demonstrated previously. The former seemed to be more prominent in unprimed BMSCs conditions, whereas the latter was accentuated in primed MSCs, however this speculation would need to be further explored in studies aimed at deciphering the intracellular molecular pathways and intercellular communication at play during the formation of the osteogenic microenvironment.

In conclusion, this study demonstrates the disparate gene expression, secreted paracrine factors, and tri-lineage differentiation potential of BMSCs and CBMSCs in vitro, despite them sharing identical MSC-specific surface markers. It also reveals that while unprimed CBMSCs do not form ectopic bone in vivo, unlike BMSCs, in vitro priming prior to transplantation may impart osteogenic capacity to CBMSCs. To the best of our knowledge, this is the first study to show the osteoinductive potential of CBMSCs as a consequence of chondrogenic priming, or indeed of BMP-4 administration. These may present important avenues for the use of CBMSCs for bone regeneration in the clinic, while still acknowledging the superior potential of BMSCs for bone repair.

## Materials and methods

### Biomaterial

Biphasic calcium phosphate (BCP) biomaterials, comprised of hydroxyapatite and beta-tricalcium phosphate in a ratio of 20:80 by weight, ranging in size from 0.5 to 1 mm, were supplied by Biomatlante (Vigneux de Bretagne, France). These materials were chosen as they have previously been successfully employed in preclinical^5,36^ and clinical trials^8,43^ as delivery vehicles for stromal cell therapy. The overall porosity (% volume) was 75% ± 5%, with a pore size distribution of 0–10 µm (70%), 10–100 µm (20%), and 100–300 µm (10%). Biomaterials were steam sterilized by autoclaving.

### Isolation and characterization of MSCs

Bone marrow (BM) aspirates were acquired from the iliac crest of healthy donors (22.66 ± 4.51 years old; mean ± standard deviation), by the standard puncture and aspiration method in heparinized syringes. BMSCs were isolated from mononuclear cells cultured at 1×10^4^ cells/cm^2^ by plastic adherence as described previously^6^. CBMSCs were isolated from mononuclear cells cultured at 1×10^6^ cells/cm^2^ by plastic adherence as previously described^44,45^. Written informed consent was obtained from all the donors involved in the study. All research was performed in accordance with relevant guidelines/regulations. Experiments involving human cells were conducted according to the amended Declaration of Helsinki. Ethics evaluation was attained by the Ethical Committee of Fondazione IRCCS Ca’ Granda Ospedale Maggiore Policlinico n° 1982, 14th January 2020. Passage 4-5 MSCs from 3 donors of BM and 3 donors of CB were used. Cells were expanded *ex vivo* by the established plastic adhesion 2D culture method. Basal culture medium consisted of αMEM GlutaMax, supplemented with 100 U/mL penicillin and 100 μg/mL streptomycin, and 20% FBS.

The morphology of MSCs was observed by fluorescent microscopy. Cells were fixed in 4% paraformaldehyde (PFA) and cell membranes were permeabilized with 0.1% Triton X-100 and 0.2% Tween in PBS followed by incubation with 1% BSA and 5% goat serum in PBS to reduce non-specific background staining. The actin cytoskeleton of cells was stained with rhodamine phalloidin (Alexa Fluor 488 Phalloidin, Invitrogen by Life Technologies, Saint Aubin, France) at a dilution of 1/40 in 1% BSA. Cell nuclei were stained with 4′,6-diamidino-2-phenylindole, dihydrochloride (DAPI) at a concentration of 1/40 000 (Molecular Probes by Life Technologies). For phenotypic analysis of MSCs, flow cytometry was performed as previously described^6^. Briefly, cells were characterized by using the following antibodies: CD73-PE, CD90-FITC, CD105-FITC, CD3-FITC, CD34-PE, and CD45-FITC; CD3 was sourced from Beckman Coulter (Paris, France), CD73 was purchased from BD Biosciences (Le Pont de Claix, France), while all others were sourced from BioLegend through Ozyme (Paris, France).

Tri-lineage differentiation capacity of BMSCs and CBMSCs was also investigated. To assess osteogenic potential, MSCs were plated at a density of 5 × 10^3^/cm^2^ in 24 well plates in basal media. After 1 day, differentiation was induced towards an osteogenic lineage by using standard osteogenic supplements (250 μM ascorbic acid, 10 mM *β*-glycerolphosphate, and 100 nM dexamethasone). Mineralization was detected after 14 and 21 days with a 40 mM alizarin red solution (pH 4.1–4.3), while extracellular alkaline phosphatase (ALP) activity was identified by staining with a mixture of naphthol AS-MX phosphate alkaline solution with fast blue RR salt (85L2-1KT; Sigma-Aldrich, Saint Louis, MI, USA). For adipogenic differentiation, MSCs were plated at a density of 2 × 10^4^/cm^2^ in 24 well plates. Cells were cultured until 80% confluent in basal media and then induced towards adipogenic lineage by using StemPro Adipogenesis Differentiation Kit (Gibco, Life Technologies, France) according to the manufacturers’ instructions. After 21 days of differentiation, cells were stained with Oil Red O solution in 2-propanol, diluted to 60% using deionized water. Chondrogenic differentiation was induced in pellet culture (0.5 × 10^6^ cells/pellet) by chondrogenic supplements (10 ng/mL TGF-b3, 50 mg/mL ascorbic acid, 4.7 mg/mL linoleic acid, 100 nM dexamethasone and 1× insulin-transferrin-selenium) for 21 days after which sections through pellets were stained with Alcian blue to identify glycosaminoglycans. Population doubling time and cumulative population doublings were assessed and calculated as described in previous studies^45,46^.

### In vitro osteogenic or chondrogenic priming of BMSCs and CBMSCs

Prior to in vivo implantations, BMSCs and CBMSCs were cultured for 6 days in either basal media or media supplemented with; (a) standard osteogenic supplements: 10 mM β-glycerolphosphate, 250 μM ascorbic acid and  10nM dexamethasone, (b) BMP-4 (50 ng/mL), or (c) chondrogenic supplements (10 ng/mL TGF-b3, 50 mg/mL ascorbic acid, 4.7 mg/mL linoleic acid, 100 nM dexamethasone and 1× insulin-transferrin-selenium). Priming with chondrogenic induction factors prior to transplantation was performed in 2D cultures, unlike in the tri-linage differentiation assay. This was performed in order to have the same culture conditions and seeding densities as the other priming conditions. 2D monolayers, while not as efficient as 3D pellet cultures at in vitro chondrogenesis, have been shown to permit chondrogenic differentiation of MSCs^47^.

### In vitro proliferation and intracellular ALP activity of BMSCs and CBMSCs

Cell number was measured by using a fluorescent Quant-iT PicoGreen dsDNA reagent assay kit (Invitrogen) according to the manufacturer's instructions. Briefly, cells were lysed in a buffer consisting of 0.1% Triton x-100, 5 mM Tris-HCl, pH 8, followed by three freeze/thaw cycles, and the quantity of double stranded DNA was measured in the supernatant of the solutions. Fluorescent intensity was quantified at 485 nm Excitation and 535 nm Emission on a microplate reader (Tristar LB 941; Berthold Technologies, Thoiry, France) and converted to ng of DNA by using a standard lambda DNA solution. Osteogenic differentiation was investigated by intracellular alkaline phosphatase (ALP) expression in cell lysates by using an ALP Colorimetric Assay Kit (Abcam). The ALP enzyme converts the *p*-nitrophenyl phosphate (*p*NPP) substrate to an equivalent quantity of coloured *p*-Nitrophenol (*p*NP). Colorimetric absorbance was measured at 405 nm on a micro-plate reader.

### Cytokine secretion by multiplex array analysis

Soluble secretions in MSC conditioned media was assessed by using Multiplex Arrays as per the manufactures guidelines (Millipore). Briefly, a Human Cytokine/Chemokine and a Human Bone Magnetic Bead Panels were employed, samples and standards were incubated with anti-immobilized beads, then with detection antibodies and streptavidin-phycoerythrin. All wash steps were performed on an automatic magnetic washer.

### Gene expression by qRT-PCR

Total RNA was isolated using RNeasy Plus Mini-kit (Qiagen, Hilden, Germany), following manufacturer’s instructions. First strand cDNA was synthesized from 800 ng of total RNA in a 20 μL final volume reaction, using the iScript cDNA synthesis kit (Bio-Rad Laboratories, Hercules, CA, USA), according to the manufacturer's instructions. Real-time qRT-PCR was carried out using SsoFast EvaGreen Supermix (Bio-Rad Laboratories) in the CFX96 real-time PCR Detection System instrument (Bio-Rad Laboratories). To confirm product specificity, a melting curve analysis was performed after each amplification. Gene expression was calculated by the ΔΔC_t_ method, using TATA-binding protein (TBP) as house-keeping gene. Bio-Rad CFX Manager software was used for expression data generation. Primer sequences will be provided upon request.

### Attachment of MSCs to biomaterial prior to implantation

Scanning electron microscopy (SEM) was used to visualize the attachment of hMSCs onto the surface of the BCP particles after 1 h, the time allowed for cell attachment prior to in vivo implantation in this study. Cells were fixed with 4% paraformaldehyde (PFA), washed with PBS and dehydrated in graded series of ethanol: 50%, 70%, 95%, 100%. Samples were then mounted on aluminium stubs, sputter coated with gold, and viewed with a SEM (Hitachi 3000, Tokyo, Japan) operating at an acceleration voltage of 5 kV. Cell attachment was also verified by methylene blue staining and a stereomicroscope (Zeiss, Stemi 2000-C, Germany). Briefly, cells were fixed in 4% PFA, rinsed in PBS and stained with 1% (w/v) methylene blue (in dH_2_0).

### Culture of MSCs on BCP biomaterial

MSCs were resuspended at a concentration of 3.75 × 10^6^ cells/mL in MSC basal medium. A volume of 80 μL of cell suspension was then seeded onto 50 mg of BCP biomaterial deposited at the bottom of 15 mL tubes (n = 4 experimental replicates each). MSCs were allowed to adhere for 4 h at 37 °C, 5% CO_2_, after which additional MSC basal medium was added. Cultures were maintained for 6 days, changing medium on day 4 to collect 48 h conditioned medium at day 6. Conditioned media was centrifuged at 300×*g* for 10 min and analysed. To take into account background from cell culture medium, the same experimental conditions were applied to BCP incubated with MSC medium only (n = 3). One out of four MSCs-BCP construct replicates was dedicated to microculture tetrazolium (MTT) assay to normalize the levels of secreted proteins to cell number. Briefly, MSCs-BCP constructs were washed with PBS, incubated with 1 mL of 0.5 mg/mL of Thiazolyl Blue Tetrazolium Bromide (Sigma-Aldrich) for 2 h at 37 °C, 5% CO_2_. Next, after another PBS washing step, MSCs-BCP constructs were incubated with 100 μL of 96% ethanol to extract the formazan crystal. The absorbance of dissolved crystals was read at 570 nm on the GENios microplate reader (TECAN, Männedorf, Switzerland) running Magellan software (TECAN).

RNA extraction was performed from three replicates for each MSC population with Trizol (Fisher Molecular Biology, Rome, Italy), following standard procedures with modifications^48^. RNA concentration and quality were verified using a NanoDrop ND-1000 spectrophotometer (NanoDrop Technologies). Retro-transcription was performed with SuperScript IV VILO Master Mix (Thermo Fisher Scientific), while qRT-PCR was carried out as described above, using Power-Up SYBR Green Master Mix (Thermo Fisher Scientific), following manufacturers’ indications.

To quantify extracellular ALP, 100 μL of conditioned medium was incubated with 1 μL of AP Live Stain (Thermo Fisher Scientific, Waltham, MA, USA) for 1 h at 37 °C. Background control medium alone or supplemented with 1–0.5–0.25 μL of 1 U/μL of Anza Alkaline Phosphatase (Thermo Fisher Scientific) was used as negative and positive controls, respectively. All samples were analysed in duplicate. Fluorescence was read on the GENios microplate reader, using 485/25 nm excitation and 535/35 nm emission filter set. To quantify IL-8 and IL-6 cytokines, pre-coated ELISA kits (Peprotech, Rocky Hill, USA) were used, following manufacturer’s instructions.

### MSC transplantation into the subcutis of nude mice

All animal experiments were conducted in keeping with the Directive 2010/63/UE and ARRIVE guidelines and after approval (CEEA.2012.199) of protocols from the local and national ethical committee (CEEA, Pays-de-la-Loire, France). 4–10 implants were prepared per group, using cells from six different human donors: three for BM and three for CB. These were the same donors as those used for in vitro experiments. BMSCs and CBMSCs in passage 4–5 with/without priming were suspended in basal media and mixed with BCP granules (2 × 10^6^ cells/50 mg BCP for each implant) for 1 h prior to implantation in order to allow cells to attach to the biomaterial. This cell/biomaterial ratio was previously determined to be optimal^4^. BCP biomaterials alone served as negative controls. Immunocompromised nude female mice (RjOrl: NMRI-*Foxn1*^*nu*^/*Foxn1*^*nu*^) at 4 weeks of age were purchased from Janvier Labs, Saint-Berthevin, France, were placed under general anaesthesia by inhalation of isoflurane. Two subcutaneous implants were performed on the dorsal side of each mouse. After 8 weeks, animals were sacrificed and implants were excised and fixed in 4% formal buffered solution.

### Histology and histomorphometry

Explants were decalcified in a PBS solution containing 4.13% EDTA/0.2% PFA, pH 7.4 at 50 °C using a decalcifying microwave apparatus (KOS Histostation, Milestone Med. Corp. USA). Samples were dehydrated in ascending series of ethanol and butanol in a dehydration station (MicromMicrotech, Lyon, France). Samples were embedded in paraffin (Histowax; Histolab, Gottenburg, Sweden) and thin sections (3 µm thick) were stained by the Masson’s trichrome technique, staining cell nuclei blue/black with hematoxylin, cytoplasm, muscle and erythrocytes red using fuchsine, and collagen green using light green solution. Sections were scanned (NanoZoomer; Hamamatsu, Photonics, Hamamatsu City, Shizuoka, Japan) and observed using a virtual microscope (NDP view; Hamamatsu). Histomorphometry of images was completed using ImageJ with the color deconvolution plugin, and percentage of bone and bone marrow were calculated per area of explants.

### Immunohistochemistry

IHC of CD146 to identify blood vessels and Vimentin to identify engrafted human cells was performed. For both protocols paraffin embedded tissue samples were cut with a microtome and adhered to poly-lysine slides, dewaxed, and rehydrated. Heat-mediated antigen retrieval was performed over night with Tris-EDTA (pH 9) at 60 °C, followed by quenching of endogenous peroxidase activity by 3% hydrogen peroxide. Nonspecific binding sites were blocked with 2% goat serum, 1% bovine serum albumin (BSA). All washing steps were conducted using T.B.S. 1× pH = 7.4 Tween 0.05%. Neovascularization was identified with the CD146 rabbit monoclonal antibody which identifies pericyte cells (ab75769; Abcam) at a dilution of 1:400 was used, followed by incubation with the goat anti-rabbit secondary antibody at a dilution of 1:400 (E0432; Dako). The engraftment of human cells at the implant site was assessed by using an antibody against human Vimentin, a protein expressed in mesenchymal cells and their derivatives. Samples were incubated with the primary antibody (Monoclonal Mouse anti-Vimentin clone V9, Dako, M0725) at a dilution of 1:800, followed by incubation with the secondary antibody (goat anti-mouse biotinylated, Dako, E0433) at a dilution of 1:500. For both protocols the target antigen signal was amplified using streptavidin peroxidase (DAKO, P0397), while diaminobenzidine (DAB) was used as the chromogen. All sections were dehydrated and counterstained using Gill’s hematoxylin and mounted using Pertex mounting medium. Sections were scanned (NanoZoomer; Hamamatsu, Photonics, Hamamatsu City, Shizuoka, Japan) and observed using a virtual microscope (NDP view; Hamamatsu). Images were processed using ImageJ and area of staining, number of blood vessel/mm^2^, and size of vessels were measured in the case of CD146 staining, while for Vimentin the number of implants with human cells present over the total number of implants was identified.

### Statistical analysis

Experiments were performed using three human donors of CBMSCs and three human donors of BMSCs. Data were expressed as mean ± standard error of the mean (SEM), where not specified otherwise. When considering differences between MSCs of each origin, where multiple time points or priming conditions were considered, statistical differences between group means were tested by using either a one-way, or two-way ANOVA, followed by Holm–Sidak’s multiple comparison test. Statistical differences between CBMSC and BMSC groups where experiments were conducted at a single time point or single experimental condition were assessed by an unpaired Student’s *t*-tests. Statistical analyses were conducted with software package GraphPad Prism. A p-value ≤ 0.05 was considered statistically significant and indicated in the figures following figure legend indications. Statistically non-significant results were specified in the text only.

## Supplementary Information

Supplementary Figure 1.

## References

[1] Pittenger MF (1999). Multilineage potential of adult human mesenchymal stem cells. Science.

[2] Zuk PA (2001). Multilineage cells from human adipose tissue: Implications for cell-based therapies. Tissue Eng..

[3] Erices A, Conget P, Minguell JJ (2000). Mesenchymal progenitor cells in human umbilical cord blood. Br. J. Haematol..

[4] Brennan MÁ (2014). Pre-clinical studies of bone regeneration with human bone marrow stromal cells and biphasic calcium phosphate. Stem Cell Res. Ther..

[5] Gamblin A-L (2014). Bone tissue formation with human mesenchymal stem cells and biphasic calcium phosphate ceramics: The local implication of osteoclasts and macrophages. Biomaterials.

[6] Brennan MA (2017). Inferior in vivo osteogenesis and superior angiogeneis of human adipose-derived stem cells compared with bone marrow-derived stem cells cultured in xeno-free conditions. Stem Cells Translat. Med..

[7] Granchi D (2017). Changes of bone turnover markers in long bone nonunions treated with a regenerative approach. Stem Cells Int..

[8] Gómez-Barrena E (2018). Feasibility and safety of treating non-unions in tibia, femur and humerus with autologous, expanded, bone marrow-derived mesenchymal stromal cells associated with biphasic calcium phosphate biomaterials in a multicentric, non-comparative trial. Biomaterials.

[9] Gjerde C (2018). Cell therapy induced regeneration of severely atrophied mandibular bone in a clinical trial. Stem Cell Res. Ther..

[10] Ragni E (2013). Adipogenic potential in human mesenchymal stem cells strictly depends on adult or foetal tissue harvest. Int. J. Biochem. Cell Biol..

[11] Wagner W (2005). Comparative characteristics of mesenchymal stem cells from human bone marrow, adipose tissue, and umbilical cord blood. Exp. Hematol..

[12] Kang X-Q (2006). Differentiating characterization of human umbilical cord blood-derived mesenchymal stem cells in vitro. Cell Biol. Int..

[13] Hutson EL, Boyer S, Genever PG (2005). Rapid isolation, expansion, and differentiation of osteoprogenitors from full-term umbilical cord blood. Tissue Eng..

[14] Park K-S, Lee Y-S, Kang K-S (2006). In vitro neuronal and osteogenic differentiation of mesenchymal stem cells from human umbilical cord blood. J. Vet. Sci..

[15] Chang Y-J (2006). Disparate mesenchyme-lineage tendencies in mesenchymal stem cells from human bone marrow and umbilical cord blood. Stem Cells.

[16] Handschel J (2010). Comparison of ectopic bone formation of embryonic stem cells and cord blood stem cells in vivo. Tissue Eng. Part A.

[17] Liu G (2010). In vitro and in vivo evaluation of osteogenesis of human umbilical cord blood-derived mesenchymal stem cells on partially demineralized bone matrix. Tissue Eng. Part A.

[18] Brocher J (2013). Inferior ectopic bone formation of mesenchymal stromal cells from adipose tissue compared to bone marrow: rescue by chondrogenic pre-induction. Stem Cell Res..

[19] Farrell E (2011). In-vivo generation of bone via endochondral ossification by in-vitro chondrogenic priming of adult human and rat mesenchymal stem cells. BMC Musculoskelet. Disord..

[20] Freeman FE (2020). A developmental engineering-based approach to bone repair: endochondral priming enhances vascularization and new bone formation in a critical size defect. Front. Bioeng. Biotechnol..

[21] Osinga R (2016). Generation of a bone organ by human adipose-derived stromal cells through endochondral ossification. Stem Cells Transl. Med..

[22] Thompson EM, Matsiko A, Kelly DJ, Gleeson JP, O’Brien FJ (2016). An endochondral ossification-based approach to bone repair: Chondrogenically primed mesenchymal stem cell-laden scaffolds support greater repair of critical-sized cranial defects than osteogenically stimulated constructs in vivo. Tissue Eng. Part A.

[23] Veronesi E (2014). Transportation conditions for prompt use of ex vivo expanded and freshly harvested clinical-grade bone marrow mesenchymal stromal/stem cells for bone regeneration. Tissue Eng. Part C Methods.

[24] Vidal L, Kampleitner C, Brennan MÁ, Hoornaert A, Layrolle P (2020). Reconstruction of large skeletal defects: Current clinical therapeutic strategies and future directions using 3D printing. Front. Bioeng. Biotechnol..

[25] Barilani M (2020). A circular RNA map for human induced pluripotent stem cells of foetal origin. EBioMedicine.

[26] Shetty P, Cooper K, Viswanathan C (2010). Comparison of proliferative and multilineage differentiation potentials of cord matrix, cord blood, and bone marrow mesenchymal stem cells. Asian J. Transfus. Sci..

[27] Rebelatto CK (2008). Dissimilar differentiation of mesenchymal stem cells from bone marrow, umbilical cord blood, and adipose tissue. Exp. Biol. Med. (Maywood).

[28] Kern S, Eichler H, Stoeve J, Klüter H, Bieback K (2006). Comparative analysis of mesenchymal stem cells from bone marrow, umbilical cord blood, or adipose tissue. Stem Cells.

[29] White JL, Walker NJ, Hu JC, Borjesson DL, Athanasiou KA (2018). A comparison of bone marrow and cord blood mesenchymal stem cells for cartilage self-assembly. Tissue Eng. Part A.

[30] Sacchetti B (2016). No identical ‘mesenchymal stem cells’ at different times and sites: Human committed progenitors of distinct origin and differentiation potential are incorporated as adventitial cells in microvessels. Stem Cell Rep..

[31] Day AGE (2018). Osteogenic potential of human umbilical cord mesenchymal stem cells on coralline hydroxyapatite/calcium carbonate microparticles. Stem Cells Int..

[32] Shirakabe K, Terasawa K, Miyama K, Shibuya H, Nishida E (2001). Regulation of the activity of the transcription factor Runx2 by two homeobox proteins, Msx2 and Dlx5. Genes Cells.

[33] Hassan MQ (2004). Dlx3 transcriptional regulation of osteoblast differentiation: temporal recruitment of Msx2, Dlx3, and Dlx5 homeodomain proteins to chromatin of the osteocalcin gene. Mol. Cell. Biol..

[34] Marciano DP (2015). Pharmacological repression of PPARγ promotes osteogenesis. Nat. Commun..

[35] Brusotti G (2017). Betulinic acid is a PPARγ antagonist that improves glucose uptake, promotes osteogenesis and inhibits adipogenesis. Sci. Rep..

[36] Humbert P (2019). Immune modulation by transplanted calcium phosphate biomaterials and human mesenchymal stromal cells in bone regeneration. Front. Immunol..

[37] Magatti M (2019). Perinatal mesenchymal stromal cells and their possible contribution to fetal–maternal tolerance. Cells.

[38] Basile M, Marchegiani F, Novak S, Kalajzic I, Di Pietro R (2020). Human amniotic fluid stem cells attract osteoprogenitor cells in bone healing. J. Cell Physiol..

[39] Tasso R (2009). Recruitment of a host’s osteoprogenitor cells using exogenous mesenchymal stem cells seeded on porous ceramic. Tissue Eng. Part A.

[40] Herrmann M, Verrier S, Alini M (2015). Strategies to stimulate mobilization and homing of endogenous stem and progenitor cells for bone tissue repair. Front. Bioeng. Biotechnol..

[41] Wiklander, O. P. B., Brennan, M. Á., Lötvall, J., Breakefield, X. O. & El Andaloussi, S. Advances in therapeutic applications of extracellular vesicles. *Sci. Transl. Med*. **11**, 492 (2019). 10.1126/scitranslmed.aav8521 PMC7104415 31092696

[42] Brennan, M. Á., Layrolle, P. & Mooney, D. J. Biomaterials functionalized with MSC secreted extracellular vesicles and soluble factors for tissue regeneration. *Adv. Funct. Mater.* **30**, 1909125 (2020). 10.1002/adfm.201909125 PMC7494127 32952493

[43] Gómez-Barrena E (2020). Early efficacy evaluation of mesenchymal stromal cells (MSC) combined to biomaterials to treat long bone non-unions. Injury.

[44] Barilani M (2016). A chemically defined medium-based strategy to efficiently generate clinically relevant cord blood mesenchymal stromal colonies. Cell Transpl..

[45] Barilani M (2015). Dissection of the cord blood stromal component reveals predictive parameters for culture outcome. Stem Cells Dev..

[46] Barilani M (2019). Central metabolism of functionally heterogeneous mesenchymal stromal cells. Sci. Rep..

[47] Oberbauer E (2016). A luciferase-based quick potency assay to predict chondrogenic differentiation. Tissue Eng. Part C Methods.

[48] Lee JTY, Tsang WH, Chow KL (2011). Simple modifications to standard TRIzol protocol allow high-yield RNA extraction from cells on resorbable materials. J. Biomater. Nanobiotechnol..

