# Supplementary material for: Chondrogenic and BMP-4 primings confer osteogenesis potential to human cord blood mesenchymal stromal cells delivered with biphasic calcium phosphate ceramics
Source: Sci Rep. 2021 Mar 24;11:6751. doi: 10.1038/s41598-021-86147-9 (PMC7991626; doi:10.1038/s41598-021-86147-9)
Supplement: Supplementary file 1 — Supplementary Figure 1. [file 41598_2021_86147_MOESM1_ESM.docx]

**Supplementary Information**

**Chondrogenic and BMP-4 primings confer osteogenesis potential to human cord blood mesenchymal stromal cells delivered with biphasic calcium phosphate ceramics**

Running title: Osteogenic potential of human umbilical cord blood and bone marrow mesenchymal stromal cells

**Authors** Meadhbh Á Brennan^1,*^, Mario Barilani^2,*^, Francesco Rusconi^2^, Julien de Lima^1^, Luciano Vidal^1^, Cristiana Lavazza^2^, Lorenza Lazzari^2^, Rosaria Giordano^2^, Pierre Layrolle^1^

**Affiliations**

^1^Inserm, UMR 1238, PHY-OS Laboratory, Bone sarcomas and remodelling of calcified tissues, Faculty of Medicine, University of Nantes, Nantes, France

^2^Laboratory of Regenerative Medicine – Cell Factory, Department of Transfusion Medicine and Hematology, Fondazione IRCCS Ca' Granda Ospedale Maggiore Policlinico, Milano, Italy

*These authors contributed equally to this work

Corresponding author: Pierre Layrolle [pierre.layrolle@inserm.fr](mailto:pierre.layrolle@inserm.fr)


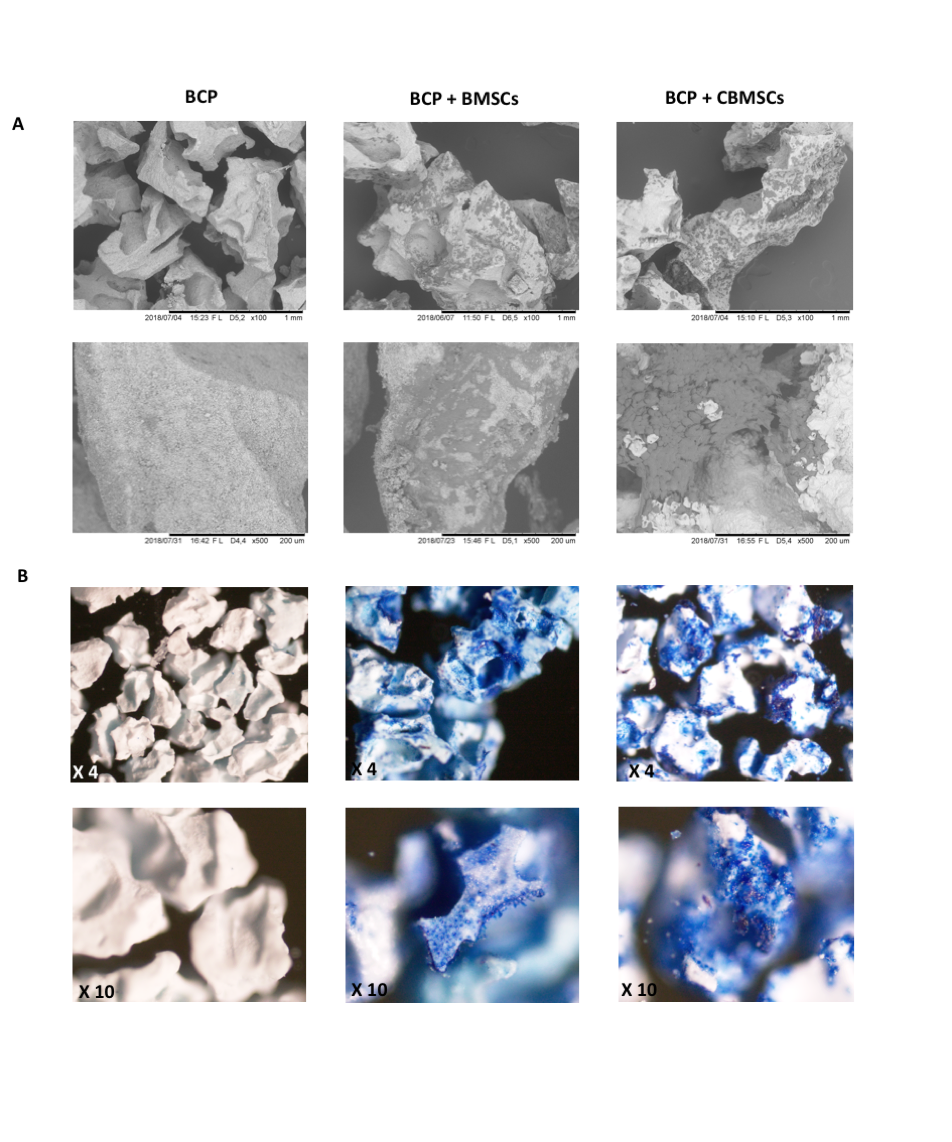


**Supplementary Figure 1. Attachment of MSCs to biomaterials. A)** Scanning Electron Microscopy images of BCP biomaterials with MSCs from either bone marrow or umbilical cord blood attached after 1 hour of incubation. Scale bar in upper panel indicates 1mm, while lower panel indicates 200 μm. **B)** Methylene blue staining of MSCs from both tissue origins showing cells (blue) attached to BCP biomaterials (white), with 4X and 10X magnification of upper and lower panels respectively.
